# Heat acclimation enhances the cold-induced vasodilation response

**DOI:** 10.1007/s00421-021-04761-x

**Published:** 2021-07-10

**Authors:** Urša Ciuha, Alexandros Sotiridis, Tinkara Mlinar, Joshua T. Royal, Ola Eiken, Igor B. Mekjavic

**Affiliations:** 1 Department of Automation, Biocybernetics and Robotics, Jozef Stefan Institute, 1000 Ljubljana, Slovenia; 2 Section of Sport Medicine and Biology of Exercise, School of Physical Education and Sport Science, National and Kapodistrian University of Athens, Athens, Greece; 3 Jozef Stefan International Postgraduate School, Ljubljana, Slovenia; 4 Department of Environmental Physiology, School of Health and Technology, Royal Institute of Technology, Stockholm, Sweden

**Keywords:** Cold-induced vasodilation, Cross-adaptation, Exercise training, Heat acclimation, Hypoxic acclimation

## Abstract

**Purpose:**

It has been reported that the cold-induced vasodilation (CIVD) response can be trained using either regular local cold stimulation or exercise training. The present study investigated whether repeated exposure to environmental stressors, known to improve aerobic performance (heat and/or hypoxia), could also provide benefit to the CIVD response.

**Methods:**

Forty male participants undertook three 10-day acclimation protocols including daily exercise training: heat acclimation (HeA; daily exercise training at an ambient temperature, *T*_a_ = 35 °C), combined heat and hypoxic acclimation (HeA/HypA; daily exercise training at *T*_a_ = 35 °C, while confined to a simulated altitude of ~ 4000 m) and exercise training in normoxic thermoneutral conditions (NorEx; no environmental stressors). To observe potential effects of the local acclimation on the CIVD response, participants additionally immersed their hand in warm water (35 °C) daily during the HeA/HypA and NorEx. Before and after the acclimation protocols, participants completed hand immersions in cold water (8 °C) for 30 min, followed by 15-min recovery phases. The temperature was measured in each finger.

**Results:**

Following the HeA protocol, the average temperature of all five fingers was higher during immersion (from 13.9 ± 2.4 to 15.5 ± 2.5 °C; *p* = 0.04) and recovery (from 22.2 ± 4.0 to 25.9 ± 4.9 °C; *p* = 0.02). The HeA/HypA and NorEx protocols did not enhance the CIVD response.

**Conclusion:**

Whole-body heat acclimation increased the finger vasodilatory response during cold-water immersion, and enhanced the rewarming rate of the hand, thus potentially contributing to improved local cold tolerance. Daily hand immersion in warm water for 10 days during HeA/Hyp and NorEx, did not contribute to any changes in the CIVD response.

## Introduction

The possibility that an optimal cold-induced vasodilation (CIVD) response may offer protection against local cold injury has provided the impetus for studies investigating the manner in which the CIVD response could be trained. Several studies observed an enhanced CIVD response in the hands of cold-acclimatised humans, including those born in cold regions or regularly exposed to other sources of cold (Nelms and Soper 1962). The hands of cold-acclimatised individuals exhibit increased blood flow, resulting in higher skin temperature, and earlier onset of the CIVD response. However, this local adaptation to a cold stimulus is greatly dependent on the thermal state of the body as a whole (Daanen 2003), with the CIVD response more pronounced when the body core and skin temperatures are elevated during local cooling (Daanen and Ducharme 1999; Daanen et al. 1997). Whole-body cold exposure, observed in Korean female divers wearing only thin cotton bathing suits, has been shown to induce insulative acclimatisation, evident in reduced heat loss during cold stress, rather than vasomotor acclimatisation, manifested in decreased susceptibility to cold pain or cold injury in the extremities (Lee et al. 2017). Accordingly, their CIVD response during hand immersion was weaker (Paik et al. 1972) than that observed in Arctic fishermen (Krog et al. 1960). In the 1970s, however, the Korean female divers started wearing thick wetsuits during diving, which conversely resulted in a reduced whole-body cold stress but maintained local (hand) cold stress. In the absence of core cooling, resulting from wearing the wetsuits, the repeated daily cold stimulus applied to the hands elicited an enhancement of the CIVD response (Lee et al. 2017). This suggests that the resultant higher core temperature during the dives augmented the CIVD response.

It has previously been reported that the CIVD response can be trained by regular local cold stimulation (Glaser and Whittow 1957) even in individuals primarily acclimatised to warm environments (Purkayastha et al. 1992). Furthermore, it can also be improved by supervised prolonged (5 days per week for 4 weeks, 20 days in total) exercise training (Keramidas et al. 2010) contributing to central cardiovascular adaptations (Whyte et al. 2008) as well as local improvement in vasodilatory functioning (Maiorana et al. 2003). Paradoxically, both repeated local (hand) cold stimulation and exercise training enhance the CIVD response; the former offering a repeated local cold stimulus and the latter a repeated central warm stimulus. Common to both scenarios is an apparent cross-adaptive effect, whereby long term and/or repeated exposure to a given environment leads not only to an increased tolerance to that specific environment, but also to gains or losses in tolerance to other adverse factors (Hale 1969). This concept was supported by numerous studies (Gibson et al. 2015; Heled et al. 2012; Hessemer et al. 1986; Lee et al. 2016; Lunt et al. 2010; Maloyan et al. 2005; Sotiridis et al. 2019a), but a recent study that recruited specifically cold-adapted elderly Korean female divers, clearly demonstrated an improvement in mechanisms, typically attributed to heat adaptation, including greater total sweat rate and warm perception thresholds in the extremities, when compared to elderly and young non-diving females (Lee et al. 2017). We reasoned that the vasodilatory response to cold might also be improved by the process of heat acclimation, long-known to improve the vasodilatory response to heat. To address this hypothesis, we took advantage of three acclimation studies that involved 10 days of controlled exercise training under repeated exposure to environmental stressors, including heat and hypoxia. Heat acclimation, established by controlled-hyperthermia (Fox et al. 1963), is known to enhance cardiovascular and thermoregulatory capacity, evident in reduced heart rate and increased cardiac output, as well as reduced core temperature concomitant to an earlier and enhanced sweating response (Nielsen et al. 1993; Patterson et al. 2004; Sotiridis et al. 2019a). Hypoxic exposure, on the other hand, induces haematological and cardiorespiratory modifications by stimulating erythropoietin production, potentially improving exercise performance (Levine and Stray-Gundersen 1997; Richalet and Gore 2008).

Whether the combinations of these stressors, including the exercise training itself, can also enhance the CIVD response was investigated in the present study.

## Methods

**Fig. 1:**
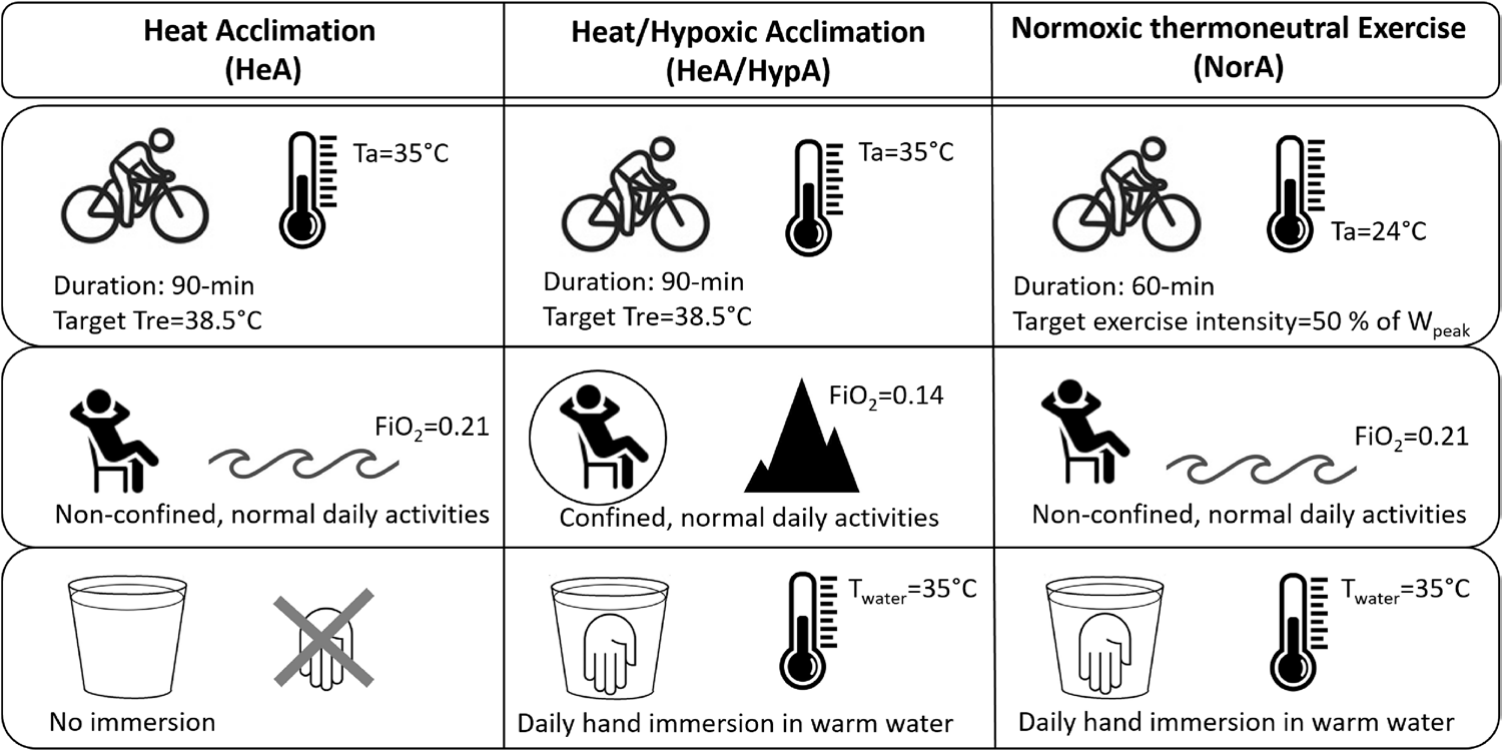
Graphical presentation of the study acclimation protocols. *T*_*re*_ rectal temperature, *T*_*a*_ ambient temperature, *HR* heart rate, *FiO*_*2*_ fraction of inspired oxygen, *T*_*water*_ water temperature during daily hand immersion

The present study was conducted within the framework of three cross-adaptation studies (Sotiridis et al. 2019a,2019b,^2020^), investigating the effects of different 10-day acclimation protocols on exercise cardiorespiratory and thermoregulatory responses. A total of 40 healthy young male participants completed the three acclimation studies, outlined in Fig. 1 (described in more detail below): (1) heat acclimation (HeA), (2) heat acclimation combined with hypoxic acclimation (HeA/HypA), and (3) normoxic thermoneutral exercise training (NorEx). The studies were performed in the summer 2017, spring 2018 and autumn 2018, respectively. Two participants were involved in two of the studies. The studies were separated from each other by more than 6 months; thus, no cross-over effect was anticipated in these two participants. The participants were healthy young males, near-sea level residents, non-smokers, with no exposure to altitude (> 1500 m) or high ambient temperatures (> 30 °C) for at least 1 month before entering the study. None of the participants had any history of cardiorespiratory or haematological diseases. They were instructed to abstain from caffeine and alcohol consumption throughout the study protocol. The experimental protocol was approved by the National Committee for Medical Ethics at the Ministry of Health (Republic of Slovenia, no. 0120-494/2018/9) and conformed to the Declaration of Helsinki guidelines.

Prior to, and following each 10-day acclimation protocol, participants’ maximal aerobic power was determined with an incremental-load exercise test on a cycle ergometer in three ambient conditions: thermoneutral normoxic conditions (ambient temperature—*T*_a_: 23.0 °C, relative humidity—RH: 50%), thermoneutral hypoxic conditions (*T*_a_: 23 °C, RH: 50%, fraction of inspired oxygen—FiO_2_: 0.14) and hot normoxic conditions (*T*_a_: 35 °C, RH: 50%). Each maximal exercise test consisted of two stages—a 30-min steady-state exercise at 40% of the preliminary normoxic peak power output (*W*_peak_), immediately followed by an incremental-load exercise to exhaustion. Following a 2-min rest period, participants commenced pedalling on the cycle ergometer. During the first 2 min of the exercise, the load was set at 90 W (warm-up). Thereafter, they cycled for 30 min at a steady-state work rate equivalent to 40% of their NOR *W*_peak_ attained during the preliminary testing. The absolute and not the relative external workload was the same in all three exercise trials (the same power output was employed for a specific participant across environmental conditions). Immediately following the 30-min steady-state exercise protocol, participants performed an incremental-load exercise to exhaustion. The initial work rate was set at 100 W (2 min), thereafter increasing by 20 W every minute until volitional exhaustion (inability to maintain the cycling cadence above 60 rpm). *W*_peak_ was calculated according to the following equation:

*W*_peak_ = work rate of last stage completed + [(work rate increment) × (time into current stage/60)]

*W*_peak_ was used to prescribe the individualised exercise training during the 10 days. Specifically, participants exercised at 50% of their maximum *W*_peak_.

### Acclimation protocols

Apart from the exercise training incorporated in all three acclimation protocols, two additional environmental stressors (heat and hypoxia) were added to the protocols to observe how different combinations would interact and affect cardiorespiratory and thermoregulatory responses (Fig. 1).

All three acclimation protocols were aiming at a central heat adaptation as a result of training and/or prolonged exposure to heat. Primarily, the idea was to observe how this central adaptation would further reflect peripherally in the CIVD response. Additionally, the study aimed to investigate the signs of local heat acclimation by immersing a hand, that was also tested for CIVD response, daily in warm water for 30 min. Since local cold exposure of the hands has been shown to augment the CIVD response (Krog et al. 1960; Nelms and Soper 1962), the hypothesis was that such benefits could also be observed after local heat exposure.

#### Heat acclimation (HeA)

Heat acclimation was achieved using the controlled-hyperthermia technique—isothermal clamping of the core temperature (Fox et al. 1963). Twelve healthy participants, ranging from moderately active to trained (*W*_peak_: 362 ± 42 W; age: 22.3 ± 2.8 years), conducted daily 90-min training sessions in a climatic chamber for 10 days in hot normoxic conditions (*T*_a_: 35 °C, RH: 50%). The 90-min training session started with a workload corresponding to 50% of participants’ thermoneutral normoxic *W*_peak_, with the target rectal temperature (*T*_re_) of 38.5 °C reached within 30–45 min of the training and maintained at the desired level for the rest of the session by adjusting power output every 5 min (± 10% *W*_peak_).

#### Heat acclimation combined with hypoxic acclimation (HeA/HypA)

Eight healthy recreationally active and moderately trained males (*W*_peak_: 343 ± 30 W; age: 23.9 ± 2.7 years) were confined to the hypoxic facility (Olympic Sports Centre Planica, Rateče, Slovenia) for 10 days at a simulated altitude of approximately 4000 m, achieved by maintaining F_i_O_2_ at 0.141 ± 0.005 in the facility situated at 940 m, resulting in an inspired partial pressure of oxygen (P_i_O_2_) of 90.4 ± 2.3 mmHg. Each day, they performed a 90-min training session in hot normoxic conditions (*T*_a_: 35 °C, RH: 50%) using the controlled-hyperthermia technique as in the HeA protocol.

#### Normoxic thermoneutral exercise training (NorEx)

Twenty males (*W*_peak_: 342 ± 58 W; age: 23.5 ± 2.6 years) completed 10 daily 60-min training sessions in normoxic thermoneutral conditions (*T*_a_: 24 °C, RH: 50%) with training intensity set at 50% of their thermoneutral normoxic *W*_peak_.

### Cold‑induced vasodilation (CIVD)

In all three studies, measurements of the CIVD response were conducted before and after each 10-day intervention (2 days before and 2 days after the protocol). Participants did not perform any exercise before the CIVD measurements were conducted and they remained seated for 20 min after entering the laboratory to adapt to the controlled ambient temperature (25 °C) and to allow us to ensure stable blood flow in the hands. Thereafter, a temperature sensor (MSR 147WD, MSR-Electronic GmbH, Switzerland) was placed on the side of each finger of the right hand attached with thin air-permeable tape (Tegaderm, 3M, USA). Once instrumented, the forearm was placed on a flat surface at the hip level for 3 min, providing the baseline measurements. Immediately after, a thin plastic bag was placed on the hand. Excess air was removed, and the bag was sealed with micropore tape (3M, USA) approximately 10 cm above the wrist. Next, the instrumented right hand was immersed up to the ulnar and radial styloid processes sequentially in tanks of warm (35 °C) and cool water (8 °C) for 5 and 30 min, respectively. During the hand immersion, participants were seated on a padded chair, with the elbow supported by a flat surface and thick towel. After the 30-min cold-water immersion, the hand was taken out of the water, the plastic bag removed, and the forearm again placed on a flat surface at the hip level for another 15 min (recovery phase). Auditory canal temperature (*T*_ac_) was measured every 5 min, using a commercial infrared thermometer (ThermoScan, Braun, Germany). Heart rate (HR) was monitored with a heart rate monitor (Polar T34, Finland) throughout the trial. CIVD measurements were conducted at the same time of the day with similar daily activities being reported pre- and post-acclimation for each participant.

To distinguish any potential benefits of the daily warm-water hand immersion, both hands were tested before and after the NorEx protocol. Both hands were immersed during the same visit, however, the immersion of each hand was separated by 30 min. Half of the participants commenced the immersion with their left hand, and the other half with the right hand. The same order of hand immersion was repeated for each participant before and after the 10-day acclimation protocol. The CIVD measurement was performed in the same manner as described above.

During the CIVD measurements, participants provided ratings of thermal comfort and thermal sensation of the immersed hand every 10 min, using the following descriptors: 0–0.5: comfortable, 1–1.5: slightly uncomfortable, 2–2.5: uncomfortable, 3: very uncomfortable for thermal comfort and 3: hot, 2: warm, 1: slightly warm, 0: neutral, − 1: slightly cool, − 2: cool and − 3: cold for thermal sensation (ANSI/ASHRAE 2013).

### Statistical analysis

The area under the curve (AUC) as an incremental area under the curve above 5 °C (Gorjanc et al. 2018), was defined and determined from the 2nd to the 30th min of the cold-water immersion, since the first 2 min were transitional with finger temperature decreasing rapidly. It was calculated from a downloadable spreadsheet—the Time Series Response Analyser (Narang et al. 2020). For other CIVD parameters, identified in previous studies (Daanen 2003; Keramidas et al. 2010; Mekjavic et al. 2008), a computer program written in LabVIEW (National Instruments, Austin, Texas) was developed. Briefly, these parameters included:

o The number of waves (N; defined as a minimum temperature increase of 0.5 °C, lasting for at least 3 min),
o Average finger temperature (*T*_avg_),
o Minimum temperature before the onset of CIVD (*T*_min_),
o Maximum temperature during CIVD (*T*_max_),
o Temperature amplitude as the difference between *T*_min_ and *T*_max_ (Δ*T*)
o Wave period as the time between two consecutive minimums (Δt)
o Onset time from immersion to the first *T*_min_ and *T*_max_ (Δt_onset_)
o Peak time as the time interval between *T*_min_ and *T*_max_ (Δt_peak_)

A paired samples *t* test was used to compare the CIVD parameters in individual fingers before and after a specific acclimation protocol. A Wilcoxon non-parametric test was used for subjective ratings. The alpha level of significance was set at 0.05. Temperature measurements are presented as means ± SD and subjective ratings as medians. Statistical analysis was performed using Statistica 8.0 (Statsoft Inc., USA).

## Results

Baseline measurements of the finger temperatures during the first 3 min of the trial did not differ before and after each of the 10-day intervention (HeA *p*: = 0.14; HeA/HypA: *p* = 0.4; NorEx: *p* = 0.3), confirming that the CIVD trials were initiated at similar finger temperatures before and after each protocol. Mean *T*_ac_ and HR remained similar before and after each acclimation protocol (Table 1).

**Table 1:** Mean heart rate (bpm; ± SD) and auditory canal temperature (°C; ± SD)

	HeA		HeA/HypA		NorEx	
	Pre	Post	Pre	Post	Pre	Post
Auditory canal temperature	36.8 ± 0.2	36.6 ± 0.3	36.7 ± 0.2	36.6 ± 0.2	36.8 ± 0.3	36.7 ± 0.2
Heart rate	72 ± 11	67 ± 10	75 ± 8	77 ± 9	73 ± 9	75 ± 8

The median rating of perceived temperature during cold-water immersion was slightly uncomfortable (from 0 to 2) and slightly cool (from 0 to − 3) with no pre- to post-intervention differences in perception observed.

### Heat acclimation (HeA)

**Fig. 2:**
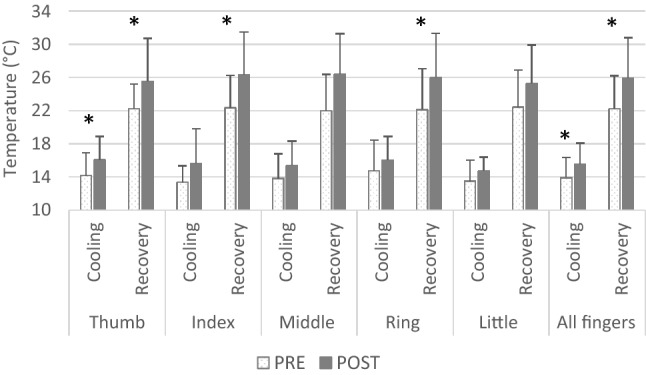
Finger temperature during hand cold-water immersion (cooling) and recovery (rewarming phase) before (white bars) and after (grey bars) the heat acclimation (HeA) protocol; 10 days of training at 35 °C with a target rectal temperature of 38.5 °C. *Indicates pre- to post-HeA significant difference (*p* < 0.05)

Following the HeA protocol, the average temperature of all five fingers was higher both during cold-water immersion (from 13.9 ± 2.4 to 15.5 ± 2.5 °C; *p* = 0.049) and during the recovery phase (from 22.2 ± 4.0 °C, to 25.9 ± 4.9 °C; *p* = 0.023). Observing the responses of individual fingers during immersion, only the thumb displayed a higher temperature post- (16.0 ± 2.8 °C) compared to pre-HeA protocol (14.2 ± 2.2 °C; *p* = 0.032), whereas the temperature of the other fingers remained similar between the trials (Fig. 2). The rewarming rate was improved in all fingers (Fig. 2), except the little finger (*p* = 0.067), although a tendency towards improved rewarming was shown. Accordingly, the AUC after the HeA protocol was greater in the thumb during cold-water immersion (from 230 ± 74 to 278 ± 81 [arbitrary units]; *p* = 0.044) and, with the exception of the little finger (from 246 ± 65 to 288 ± 66; *p* = 0.069), in all remaining fingers during the recovery phase (thumb: from 243 ± 42 to 291 ± 74, *p* = 0.026; index: from 245 ± 57 to 303 ± 73, *p* = 0.016; middle: from 239 ± 63 to 304 ± 69, *p* = 0.014; ring finger: from 242 ± 71 to 297 ± 77, *p* = 0.044; [arbitrary units]).

The analysis of CIVD parameters indicated greater number of waves after the HeA protocol, observed in the thumb (from 1.4 ± 1.1 to 2.3 ± 1.3; *p* = 0.025), index (from 1.5 ± 0.9 to 2.1 ± 1.3; *p* = 0.046) and middle finger (from 1.5 ± 0.9 to 2.3 ± 1.2; *p* ˂ 0.001). *T*_max_ in the thumb increased from 14.1 ± °C to 17.8 ± 3.3 °C after the HeA protocol (*p* = 0.010). The onset time from immersion to the first *T*_min_ and *T*_max_ in the little finger was reduced by ~ 4 min after the HeA protocol (*p* ˂ 0.05).

### Combined heat and hypoxic acclimation (HeA/HypA)

**Fig. 3:**
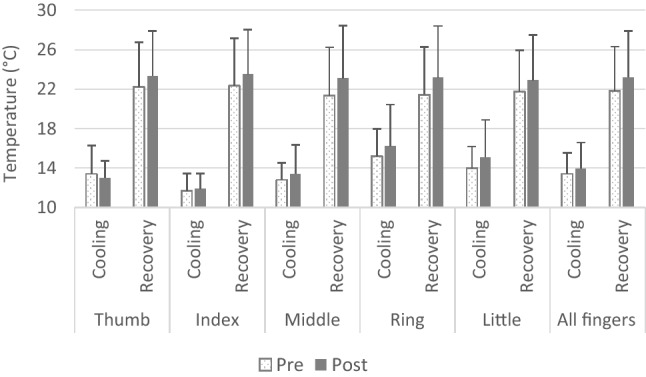
Finger temperature during the hand cold-water immersion (cooling) and recovery (rewarming phase) before (white bars) and after (grey bars) the combined heat and hypoxic acclimation (HeA/HypA) protocol: 10 days of training at 35 °C with a target rectal temperature of 38.5 °C while confined at a simulated altitude of ~ 4000 m

The average temperature of all five fingers remained unaltered during the cold-water immersion (before: 13.4 ± 2.1 °C, after: 13.9 ± 2.6 °C; *p* = 0.491) as well as the recovery phase (before: 21.8 ± 4.5 °C, after: 23.2 ± 4.7 °C; *p* = 0.491) after the combined HeA/HypA protocol. None of the individual fingers displayed pre- to post-HeA/HypA difference (Fig. 3). AUC remained unaltered in all of the fingers.

After the HeA/HypA protocol, the number of waves increased in the thumb (from 1.0 ± 0.9 to 1.8 ± 0.7; *p* = 0.048) and middle finger (from 1.1 ± 1.2 to 2.0 ± 0.8; *p* = 0.041). No other differences in any of the recorded CIVD parameters were noted.

### Normoxic thermoneutral exercise training (NorEx)

**Fig. 4:**
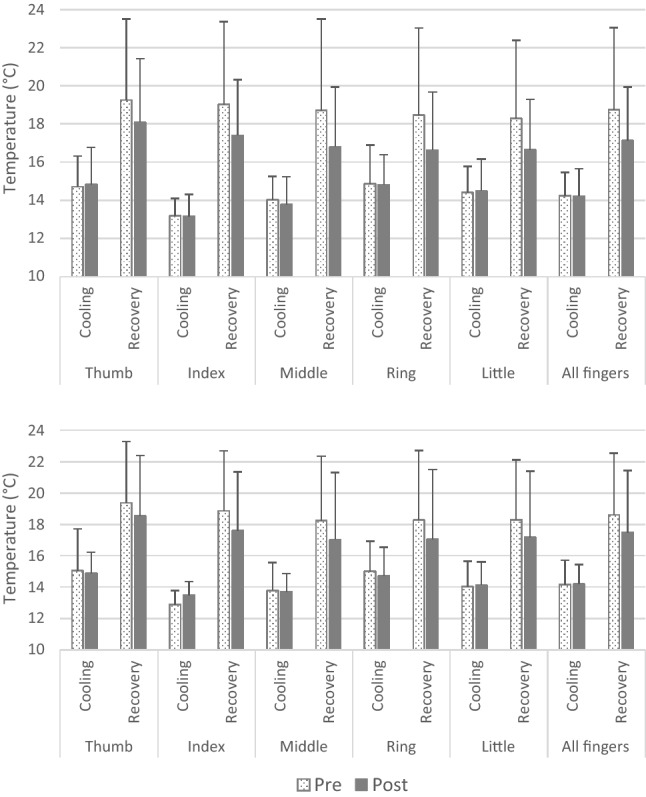
Finger temperature during the left (upper figure) and right (lower figure) hand cold-water immersion (cooling) and recovery (rewarming phase) before (white bars) and after (grey bars) the normoxic thermoneutral exercise training (NorEx) protocol: 10 days of training at 24 °C at 50% of peak power output

During the cold-water immersion, the average finger temperature of both hands did not differ between the pre- (left hand: 14.2 ± 1.2 °C, right hand: 14.1 ± 1.6 °C) and post-NorEx protocol (left hand: 14.2 ± 1.4 °C, right hand: 14.2 ± 1.2 °C; *p* = 0.933). Furthermore, during the recovery phase, the average finger temperature of both hands remained similar before (left hand: 18.7 ± 4.3 °C, right hand: 18.6 ± 3.9 °C) and after the applied protocol (left hand: 17.1 ± 2.8 °C, right hand: 17.5 ± 4.0 °C; *p* = 0.125). Individual fingers did not display any pre- to post-acclimation differences in the measured temperature (Fig. 4). AUC remained unaltered in all of the fingers.

The number of waves did not differ in any of the left and right-hand fingers when comparing pre- to post-NorEx data. The average number of waves was less than one per finger (more precisely, the average was 0.7 ± 0.6 for the left hand and 0.7 ± 0.6 for the right hand), with several participants not displaying any CIVD response before and/or after the NorEx protocol. The analysis considered only the participants, who displayed CIVD response both before and after the protocol.

Left hand: the analysis was limited to 7, 8 and 9 participants with CIVD response in the thumb, little finger and remaining fingers, respectively. Based on the available data, it was shown that the temperature amplitude (Δ*T*: the difference between *T*_min_ and *T*_max_) was actually reduced in some of the fingers following the application of the protocol, including the middle (from 2.9 ± 1.5 to 1.7 ± 1.0 °C; *p* = 0.003), ring (from 2.4 ± 1.3 to 1.7 ± 1.0 °C; *p* = 0.041) and little finger (from 2.8 ± 1.6 to 1.5 ± 0.6 °C; *p* = 0.021). The onset time from immersion to the first *T*_min_ was shorter after NorEx, observed in the index (from 16 ± 4 to 11 ± 4 min; *p* = 0.013) and ring finger (from 16 ± 3 to 11 ± 4 min; *p* = 0.021). The latter also displayed shorter time to *T*_max_ after NorEx (from 22 ± 5 to 17 ± 5 min; *p* = 0.035).

Right hand: there were only 2 participants with an evident thumb CIVD response. The data for this finger were, therefore, not considered for analysis. There were 8 participants with CIVD responses in the index and middle finger, whereas the ring and little finger displayed CIVD response in 10 and 9 participants, respectively. Further analysis revealed no differences in the measured CIVD parameters before and after NorEx in any of the fingers.

## Discussion

The present study compared the efficacy of three acclimation protocols in enhancing the CIVD response. The protocols comprised of a 10-day exercise training in combination with environmental stressors, including heat and hypoxia. During the exercise training, HeA and HeA/HypA protocols exerted similar heat stimuli to the skin and core, with the HeA/HypA protocol also including daily warm-water hand immersion. Despite this additional thermal stimuli, the CIVD response remained unchanged. In contrast, the HeA protocol exhibited an augmentation of the CIVD response, albeit not including daily warm-water hand immersion. Most likely this was due to the potential inhibitory effect of the hypoxic stimuli present in the HeA/HypA protocol. Similarly, the contribution of the NorEx protocol did not appear to have any effect on the CIVD response, although it did include daily warm-water hand immersion. Therefore, the principal finding of the study was that the CIVD response was improved by the HeA protocol, consisting of 10 daily sessions of 90-min exercise training in a warm environment (35 °C) with the participants being exposed to thermoneutral normoxic conditions for the remainder of each day. This whole-body (central) HeA seems to be more efficient than local hand acclimation as attempted by 10 daily hand immersion sessions in warm water (35 °C).

### Effect of central acclimation

As evident from the present results, the CIVD response was improved following the HeA protocol, whereas no such modifications were noted after the HeA/HypA protocol. Both studies used an identical exercise training protocol in a warm environment with the difference being that the participants in the HeA protocol were not confined and lived at an altitude of 295 m (in Ljubljana, Slovenia). The participants in the HeA/HypA protocol were confined in normobaric hypoxic conditions (a simulated altitude of ~ 4000 m) for the duration of the 10-day protocol with the 90-min exercise training protocol performed in normobaric normoxic conditions (the hypoxic facility is located at a natural altitude of 940 m). As participants in the HeA/HypA were confined to normobaric hypoxia, the lower FiO_2_ (0.141 ± 0.005) in that protocol might have inhibited heat training-anticipated benefits on the CIVD response. Indeed, these findings are in line with thermoregulatory outcomes following steady-state and incremental exercise to exhaustion (Sotiridis et al. 2019a,2019b). After the HeA protocol, our participants demonstrated both lower resting and exercise *T*_re_ as well as lower *T*_re_ thresholds for sweating and vasodilation (Sotiridis et al. 2019a), exhibiting adaptations pertinent to a classic heat adaptation phenotype (Patterson et al. 2004; Sawka et al. 2011). After the HeA/HypA protocol, only partial adaptations to heat acclimation were observed (Sotiridis et al. 2019b). It has, however, previously been reported that the CIVD response can be enhanced after a sleep high–train low regimen. The study by Amon et al. (2012) assessed the CIVD response before and after 28 days of aerobic training with daily 1-h exercise (50% of *W*_peak_) in thermoneutral conditions with the experimental group sleeping (9–12 h per night) at a simulated altitude of 2800 m (week 1) to 3400 m (week 4) and the control group sleeping close to sea level. Despite an enhanced aerobic performance post-intervention observed in both groups, the enhancement in the CIVD response was more profound in the experimental group. Some field studies have also demonstrated enhanced CIVD response in alpinists following high-altitude Himalayan expeditions (Felicijan et al. 2008; Gorjanc et al. 2018). While the study by Amon et al. (2012) was a controlled laboratory study with the hypoxic exposure limited to night time, the studies by Felicijan et al. (2008) and Gorjanc et al. (2018) were field studies involving continuous hypoxic exposure. Common to these studies is the long-term exposure (≥ 3 weeks) to simulated (normobaric hypoxia)/actual (hypobaric hypoxia) altitude and its acclimatisation effect on CIVD, which was initially observed by Mathew et al. (1977) and later explored by Daanen and van Rujten (2000). The authors observed a reduction in the CIVD response during the first weeks of high-altitude exposure, which was restored following the acclimatisation (~ 3 weeks). The acclimatisation to altitude could further explain the augmented CIVD response after three or more weeks of altitude exposure (Amon et al. 2012; Felicijan et al. 2008; Gorjanc et al. 2018). Although in the study by Amon et al. (2012) the simulated altitude was limited only to night time with no cold acclimatisation commonly experienced in the high-altitude field studies, the hypoxic exposure alone seemed to be sufficient to induce changes in the CIVD response. On the contrary, the hypoxic exposure in our HeA/HypA study might have been too short to affect CIVD, but might also have blunted the thermoregulatory benefits of the HeA protocol (Sotiridis et al. 2019a).

The exercise training itself, performed without any environmental stressors (NorEx) elicited minor benefits in the CIVD response. Mean finger temperature and the number of waves remained unaltered following the applied supervised exercise training protocol. The average number of waves was less than one per finger before and after the experimental intervention with a great number of participants displaying no CIVD response in one or more fingers. Some of the fingers of the left hand, in fact, exhibited reduced temperature amplitude after acclimation. The index and ring fingers were the only fingers displaying some improvements, evident in a shorter time interval required to reach *T*_min_ and/or *T*_max_. Previous research (Keramidas et al. 2010) has shown an increase in average finger skin temperature and the number of waves after 4 weeks of exercise training in thermoneutral conditions (1 h daily, 5 days per week, at 50% of *W*_peak_). Exercise training significantly increased VO_2peak_, whereas the potential thermoregulatory gains were not reported. Similarly, our NorEx protocol with 10-day exercise training exhibited an improvement in V̇O_2peak_ in less fit individuals. While the peak sweat rate increased in both groups when performing submaximal aerobic exercise in the heat, sweating was initiated at lower *T*_re_ only in the more fit participants. No other thermoregulatory benefits of the acclimation protocol were noted (Sotiridis et al. 2020). As such, the improvements in performance and minor thermoregulatory gains were evidently not sufficient to provoke CIVD enhancement in the present study. While in the study by Keramidas et al. (2010) participants trained for 20 days with intermittent breaks during weekends, our participants trained for 10 consecutive days. Additionally to the enhanced aerobic capacity, the longer training protocol might have further provided thermoregulatory benefits reflected in an augmented CIVD response as in the study by Keramidas et al. (2010).

### Effect of local heat acclimation

During the 10 days of HeA/HypA and NorEx protocols, participants immersed their right hand in warm water (35 °C) before attending daily exercise training. This concomitant local hand-heat acclimation was introduced to observe potential effects of such local exposure on the CIVD response. To distinguish between local and central acclimation, CIVD measurements were performed on both hands before and after the NorEx protocol. The results indicated no differences in the temperature responses between the left and right hand, meaning that the daily hand immersion in warm water did not induce any local thermoregulatory adaptations. The CIVD response also remained unchanged following the HeA/HypA protocol, confirming no effect of the local HeA protocol. It is well documented that the CIVD response is enhanced in people whose hands are regularly exposed to cold (Krog et al. 1960; Nelms and Soper 1962; Purkayastha et al. 1992). On the contrary, some laboratory studies show no such trainability of the response (Daanen et al. 2012; Mekjavic et al. 2008). A valid argument might be that studies, targeting specific groups and occupations regularly exposed to local cold stress, are actually recruiting people demonstrating a higher level of local cold tolerance, whereas individuals who experience severe negative physiological or psychological reactions to local cold exposure will likely not engage in such occupations. As such, the observed differences may not be due to an acute or chronic acclimatisation response, but rather due to pre-existing innate physiological differences (Cheung and Daanen 2012). Nonetheless, the study on Korean female divers, who daily harvest the sea bed in cold waters, provides some intriguing findings. The divers’ CIVD response was improved once they started wearing thick wetsuits, which reduced the cold stress experienced by the divers from the whole-body to local cold stress (Lee et al. 2017). Conversely, the present study observed an enhanced CIVD response as a consequence of a whole-body heat stress (training in a warm environment at 35 °C), whereas the daily hand immersion in warm water (35 °C) might not have provided the participants with a stimulus strong enough to induce local heat acclimation. Warmer water, longer daily exposures (more than 30 min per day) and longer protocols (higher number of total acclimation sessions) might modify the outcome in future investigations.

### Recovery phase following the CIVD measurement

When conducting CIVD measurements, interest is usually focussed on the finger skin temperature during cold-water immersion (Daanen et al. 2012; Keramidas et al. 2010; Mekjavic et al. 2008). However, some studies also consider the recovery phase that is initiated with the rewarming of the hand once it is removed from the cold water (Kingma et al. 2019; Ko et al. 2020; Lee et al. 2013; Keramidas et al. 2019). The present study employed a 15-min recovery phase after cold-water immersion to observe potential alterations in rewarming of the hand as a result of the acclimation protocol. Following the HeA protocol, a significant increase in finger temperature was observed during both cold-water immersion and the recovery phase. Conversely, no enhancement in the CIVD response during cold-water immersion might further explain our inability to observe improvements in rewarming of the fingers following both the HeA/HypA and NorEx protocols. Similarly, Ko et al. (2020) observed that higher minimum finger temperature during cold-water immersion is associated with a faster recovery during the rewarming phase. Cold injury may not only be attributed to tissue cooling but also to the lack of recovery following the cold exposure. Thus, future studies aiming to investigate the vasomotor response to cold should also include an assessment of that response during the rewarming/recovery phase.

### Variability in CIVD responses

In the present study, a variety of CIVD patterns was observed among different participants as well as individual fingers. The CIVD response is often presented as a typical skin cooling response interspersed with slight elevations in skin temperature. The present study confirms the atypical nature of the CIVD, as previously emphasised by Mekjavic et al. (2008). Throughout the three acclimation protocols of the present study, several types of responses were observed, such as no CIVD, small wave, big wave, early plateau, late plateau, a quick first wave and double wave. Interestingly, following the HeA and HeA/HypA protocols, four participants exhibited a profound increase in finger skin temperature during cold-water immersion or recovery. In contrast to HeA, the HeA/HypA protocol exhibited no overall significant increase in the CIVD response. Still, half of the participants notably increased the average finger skin temperature following the protocol. These individual cases suggest that combined HeA/HypA protocol was beneficial for some participants, but might require a longer adaptation protocol to achieve more consistent responses. Due to the small sample size, the current study may have been underpowered. The outcome might have been different if the number of participants was greater. Following the NorEx protocol, no such extreme cases were noted. During the recovery phase, a substantial number of participants exhibited a reduction in the mean finger skin temperature by more than ~ 2 °C (six participants in the fingers of the left hand, and five participants in the fingers of the right hand) whereas only a small number of participants improved the rewarming of the fingers after the NorEx protocol (three participants in the left and two participants in the right-hand fingers). This outcome was somewhat surprising as exercise training alone has been shown to enhance the CIVD response (Keramidas et al. 2010). As explained earlier, a longer exercise training protocol with intermittent breaks might have provided a different, more beneficial outcome.

The CIVD measurements were performed on all fingers of either the right (HeA, HeA/HypA) or both hands (NorEx). In general, fingers displayed different temperatures, number of waves as well as individual CIVD parameters. These different responses, observed in individual fingers, suggest that CIVD should be measured in all fingers, as opposed to past studies conducting the measurements on a single finger (Daanen and Van Ruiten 2000; Lee et al. 2013; O'Brien et al. 2015). It has been demonstrated that the CIVD response is more pronounced following finger immersion and reduced after hand or forearm immersion (Ducharme et al. 2001; Sendowski et al. 1997). Nonetheless, a single-digit cold-water immersion might not reflect the responses of the remaining fingers. Based on previous work (Mekjavic et al. 2013), we concluded that the CIVD responses observed during immersion in 8 °C water were not statistically different from those observed during immersion in 5 °C water. Similarly, Tyler et al. (2015) observed the CIVD response of a finger to 0 and 8 °C water immersion and concluded that both temperatures induced a CIVD response with the latter more suitable in optimising the number of CIVD waves. The participants also reported the immersion in 8 °C water as being much less uncomfortable than immersion in 5 °C water (Mekjavic et al. 2013). There being no further gain anticipated by immersions in water temperatures lower than 8 °C, this water temperature was used in the present study, and has also been used in previous studies investigating hand cold-water immersion (Gorjanc et al. 2018; Cheung and Mekjavic 2007; Daanen et al. 1997).

### Limitations and study considerations

The present study conducted acclimation protocols (HeA, HeA/HypA, NorEx) in different seasons—the summer 2017, spring 2018 and autumn 2018. Some (Ihzuka et al. 1986; Kakitsuba et al. 2011; van Ooijen et al. 2004), but not all (Ciuha et al. 2020) thermoregulatory responses can vary seasonally. Moreover, it has been shown that CIVD can vary greatly between summer and winter (Tanaka 1971). Since none of the acclimation protocols were conducted in the winter, the seasonal differences were at least partly accounted for. Furthermore, the CIVD measurements were initiated at similar core and skin temperatures to minimise any potential seasonal effects. The daily hand immersion in warm water, investigating potential local heat acclimation, was introduced only during the HeA/HypA and NorEx protocols. Unfortunately, due to logistical reasons, we were not able to conduct the local heating in the HeA protocol. Nonetheless, to distinguish between the local and central heat acclimation, the NorEx protocol was the crucial one, performing exercise training in thermoneutral conditions with no external thermal stressor, showing no changes in the CIVD response, and therefore, no benefits of this local heating. The CIVD response was evaluated by measuring the skin temperature of our participants’ fingers. Whether human feet would display a similar response remains unclear. Considering previous research work, a different outcome might be expected (Cheung and Mekjavic 2007; Daanen et al. 2012). Therefore, the future work should also consider evaluating the CIVD response of the human feet.

### Conclusion

The present study demonstrated that whole-body heat acclimation increased the vasodilatory response during cold-water immersion and enhanced the rewarming rate of the hand, thus potentially contributing to improved cold tolerance. The increased vasodilatory response was a result of a heat acclimation protocol, which has previously been shown to enhance the vasodilatory response. Whether the present results can be attributed to cross-adaptation warrants further investigation. The relative contributions of central and peripheral factors to CIVD remain unresolved. The results of the present study would suggest that whole-body heat acclimation increased the CIVD response, whereas daily immersion of the hand in warm water did not. This would favour the contribution of central over peripheral factors to the observed response.

## Data Availability

N/A.

## Abbreviations

ANOVA: Analysis of variance
AUC: Area under the curve
CIVD: Cold-induced vasodilation
FiO_2_: Fraction of inspired oxygen
HeA: Heat acclimation
HeA/HypA: Combined heat and hypoxic acclimation
HR: Heart rate
NorEx: Normoxic thermoneutral exercise training
PiO_2_: Partial pressure of oxygen
RH: Relative humidity
*T*_a_: Ambient temperature
*T*_avg_: Average finger temperature
*T*_min_: Minimum temperature before the onset of CIVD
*T*_max_: Maximum temperature during CIVD
*T*_re_: Rectal temperature
*T*_ac_: Auditory canal temperature
*T*_water_: Water temperature
*W*_peak_: Peak power output
Δ*T*: Temperature amplitude as the difference between *T*_min_ and *T*_max_
Δt: Wave period as the time between two consecutive minimums
Δt_onset_: Onset time from immersion to the first *T*_min_ and *T*_max_
Δt_peak_: Peak time as the time interval between *T*_min_ and *T*_max_

## References

[CR1] Amon M, Keramidas ME, Kounalakis SN, Mekjavic IB (2012). The effect of a sleep high-train low regimen on the finger cold-induced vasodilation response. High Alt Med Biol.

[CR2] ANSI/ASHRAE (2013). Standard 55. Thermal Environmental Conditions for Human Occupancy.

[CR3] Cheung SS, Daanen HA (2012). Dynamic adaptation of the peripheral circulation to cold exposure. Microcirculation.

[CR4] Cheung SS, Mekjavic IB (2007). Cold-induced vasodilatation is not homogenous or generalizable across the hand and feet. Eur J Appl Physiol.

[CR5] Ciuha U, Kounalakis S, McDonnell AC, Mekjavic IB (2020). Seasonal variation of temperature regulation: do thermoregulatory responses “spring” forward and “fall” back?. Int J Biometeorol.

[CR6] Daanen H (2003). Finger cold-induced vasodilation: a review. Eur J Appl Physiol.

[CR7] Daanen H, Ducharme MB (1999). Finger cold-induced vasodilation during mild hypothermia, hyperthermia and at thermoneutrality. Aviat Space Environ Med.

[CR8] Daanen HA, Van Ruiten HJ (2000). Cold-induced peripheral vasodilation at high altitudes-a field study. High Alt Med Biol.

[CR9] Daanen H, Van de Linde F, Romet T, Ducharme M (1997). The effect of body temperature on the hunting response of the middle finger skin temperature. Eur J Appl Physiol.

[CR10] Daanen HA, Koedam J, Cheung SS (2012). Trainability of cold induced vasodilatation in fingers and toes. Eur J Appl Physiol.

[CR11] Ducharme M, Greif R, Sessler D, Doufas A, Mokhtarani M (2001) Forearm tissue temperature and the CIVD response. Proc Aust Physiol Pharmacol Soc Soc 32(2) [Suppl.] 1:27P

[CR12] Felicijan A, Golja P, Milčinski M, Cheung SS, Mekjavic IB (2008). Enhancement of cold-induced vasodilatation following acclimatization to altitude. Eur J Appl Physiol.

[CR13] Fox R, Goldsmith R, Kidd D, Lewis H (1963). Acclimatization to heat in man by controlled elevation of body temperature. J Physiol.

[CR14] Gibson OR, Turner G, Tuttle JA, Taylor L, Watt PW, Maxwell NS (2015). Heat acclimation attenuates physiological strain and the HSP72, but not HSP90α, mRNA response to acute normobaric hypoxia. J Appl Physiol.

[CR15] Glaser E, Whittow G (1957). Retention in a warm environment of adaptation to localized cooling. J Physiol.

[CR16] Gorjanc J, Morrison SA, McDonnell AC, Mekjavic IB (2018). Koroška 8000 Himalayan expedition: digit responses to cold stress following ascent to Broadpeak (Pakistan, 8051 m). Eur J Appl Physiol.

[CR17] Hale HB (1969). Cross-adaptation. Environ Res.

[CR18] Heled Y, Peled A, Yanovich R, Shargal E, Pilz-Burstein R, Epstein Y, Moran DS (2012). Heat acclimation and performance in hypoxic conditions. Aviat Space Environ Med.

[CR19] Hessemer V, Zeh A, Brück K (1986). Effects of passive heat adaptation and moderate sweatless conditioning on responses to cold and heat. Eur J Appl Physiol.

[CR20] Ihzuka H, Hori S, Akamatsu T (1986). Seasonal variations of physiological responses to heat of subtropical and temperate natives. Int J Biometeorol.

[CR21] Kakitsuba N, Mekjavic IB, Katsuura T (2011). The effect of season and light intensity on the core interthreshold zone. J Physiol Anthropol.

[CR22] Keramidas ME, Musizza B, Kounalakis SN, Mekjavic IB (2010). Enhancement of the finger cold-induced vasodilation response with exercise training. Eur J Appl Physiol.

[CR23] Keramidas ME, Kölegård R, Mekjavic IB, Eiken O (2019). Interactions of mild hypothermia and hypoxia on finger vasoreactivity to local cold stress. Am J Physiol Regul Integr Comp Physiol.

[CR24] Kingma C, Hofman I, Daanen H (2019). Relation between finger cold-induced vasodilation and rewarming speed after cold exposure. Eur J Appl Physiol.

[CR25] Ko Y, Seol S-H, Kim GH, Yu HH, Lee J-Y (2020). Effects of cold exposure discontinuation on finger cold-induced vasodilation of older retired Korean female divers ‘Haenyeos’. J Therm Biol.

[CR26] Krog J, Folkow B, Fox R, Andersen KL (1960). Hand circulation in the cold of Lapps and North Norwegian fishermen. J Appl Physiol.

[CR27] Lee J-Y, Bakri I, Matsuo A, Tochihara Y (2013). Cold-induced vasodilation and vasoconstriction in the finger of tropical and temperate indigenes. J Therm Biol.

[CR28] Lee BJ, Miller A, James RS, Thake CD (2016). Cross acclimation between heat and hypoxia: heat acclimation improves cellular tolerance and exercise performance in acute normobaric hypoxia. Front Physiol.

[CR29] Lee J-Y, Park J, Kim S (2017). Cold adaptation, aging, and Korean women divers haenyeo. J Physiol Anthropol.

[CR30] Levine BD, Stray-Gundersen J (1997). “Living high-training low”: effect of moderate-altitude acclimatization with low-altitude training on performance. J Appl Physiol.

[CR31] Lunt HC, Barwood MJ, Corbett J, Tipton MJ (2010). ‘Cross-adaptation’: habituation to short repeated cold-water immersions affects the response to acute hypoxia in humans. J Physiol.

[CR32] Maiorana A, O’Driscoll G, Taylor R, Green D (2003). Exercise and the nitric oxide vasodilator system. Sports Med.

[CR33] Maloyan A, Eli-Berchoer L, Semenza GL, Gerstenblith G, Stern MD, Horowitz M (2005). HIF-1α-targeted pathways are activated by heat acclimation and contribute to acclimation-ischemic cross-tolerance in the heart. Physiol Genomics.

[CR34] Mathew L, Purkayastha S, Selvamurthy W, Malhotra M (1977). Cold-induced vasodilatation and peripheral blood flow under local cold stress in man at altitude. Aviat Space Environ Med.

[CR35] Mekjavic IB, Dobnikar U, Kounalakis SN, Musizza B, Cheung SS (2008). The trainability and contralateral response of cold-induced vasodilatation in the fingers following repeated cold exposure. Eur J Appl Physiol.

[CR36] Mekjavic IB, Dobnikar U, Kounalakis SN (2013). Cold-induced vasodilatation response in the fingers at 4 different water temperatures. Appl Physiol Nutr Metab.

[CR37] Narang BJ, Atkinson G, Gonzalez JT, Betts JA (2020). A tool to explore discrete-time data: the time series response analyser. Int J Sport Nutr Exerc Metab.

[CR38] Nelms JD, Soper DJG (1962). Cold vasodilatation and cold acclimatization in the hands of British fish filleters. J Appl Physiol.

[CR39] Nielsen B, Hales J, Strange S, Christensen NJ, Warberg J, Saltin B (1993). Human circulatory and thermoregulatory adaptations with heat acclimation and exercise in a hot, dry environment. J Physiol.

[CR40] O'Brien C, Castellani JW, Muza SR (2015). Acute hypobaric hypoxia effects on finger temperature during and after local cold exposure. High Alt Med Biol.

[CR41] Paik K, Kang B, Han D, Rennie D, Hong S (1972). Vascular responses of Korean ama to hand immersion in cold water. J Appl Physiol.

[CR42] Patterson MJ, Stocks JM, Taylor NA (2004). Humid heat acclimation does not elicit a preferential sweat redistribution toward the limbs. Am J Physiol Regul Integr Comp Physiol.

[CR43] Purkayastha S, Selvamurthy W, Ilavazhagan G (1992). Peripheral vascular response to local cold stress of tropical men during sojourn in the Arctic cold region. Jpn J Physiol.

[CR44] Richalet JP, Gore CJ (2008). Live and/or sleep high: train low, using normobaric hypoxia. Scand J Med Sci Sports.

[CR45] Sawka MN, Leon LR, Montain SJ, Sonna LA (2011). Integrated physiological mechanisms of exercise performance, adaptation, and maladaptation to heat stress. Compr Physiol.

[CR46] Sendowski I, Savourey G, Besnard Y, Bittel J (1997). Cold induced vasodilatation and cardiovascular responses in humans during cold water immersion of various upper limb areas. Eur J Appl Physiol.

[CR47] Sotiridis A, Debevec T, Ciuha U, Eiken O, Mekjavic IB (2019). Heat acclimation does not affect maximal aerobic power in thermoneutral normoxic or hypoxic conditions. Exp Physiol.

[CR48] Sotiridis A, Miliotis P, Ciuha U, Koskolou M, Mekjavic IB (2019). No ergogenic effects of a 10-day combined heat and hypoxic acclimation on aerobic performance in normoxic thermoneutral or hot conditions. Eur J Appl Physiol.

[CR49] Sotiridis A, Debevec T, Ciuha U, McDonnell AC, Mlinar T, Royal JT, Mekjavic IB (2020). Aerobic but not thermoregulatory gains following a 10-day moderate-intensity training protocol are fitness level dependent: a cross-adaptation perspective. Physiol Rep.

[CR50] Tanaka M (1971). Experimental studies on human reaction to cold. Differences in the vascular hunting reaction to cold according to sex, season and environmental temperature. Bull Tokyo Med Dent Univ.

[CR51] Tyler CJ, Reeve T, Cheung SS (2015). Cold-induced vasodilation during single digit immersion in 0 C and 8 C water in men and women. PLoS ONE.

[CR52] van Ooijen AMJ, van Marken Lichtenbelt WD, van Steenhoven AA, Westerterp KR (2004). Seasonal changes in metabolic and temperature responses to cold air in humans. Physiol Behav.

[CR53] Whyte G, George K, Shave R, Middleton N, Nevill A (2008). Training induced changes in maximum heart rate. Int J Sports Med.

